# Prevention and Potential Treatment Strategies for Respiratory Syncytial Virus

**DOI:** 10.3390/molecules29030598

**Published:** 2024-01-25

**Authors:** Bo-Wen Sun, Peng-Peng Zhang, Zong-Hao Wang, Xia Yao, Meng-Lan He, Rui-Ting Bai, Hao Che, Jing Lin, Tian Xie, Zi Hui, Xiang-Yang Ye, Li-Wei Wang

**Affiliations:** 1 School of Pharmacy, Hangzhou Normal University, Hangzhou 311121, China; 2022112025021@stu.hznu.edu.cn (B.-W.S.); 2022112025058@stu.hznu.edu.cn (P.-P.Z.); 2023112025012@stu.hznu.edu.cn (Z.-H.W.); 2023112025029@stu.hznu.edu.cn (X.Y.); 2023112025027@stu.hznu.edu.cn (M.-L.H.); 2023112025103@stu.hznu.edu.cn (R.-T.B.); 2022112025020@stu.hznu.edu.cn (H.C.); xbs@hznu.edu.cn (T.X.); huizi781@163.com (Z.H.); 2 Key Laboratory of Elemene Class Anti-Cancer Chinese Medicines, Engineering Laboratory of Development and Application of Traditional Chinese Medicines, Collaborative Innovation Center of Traditional Chinese Medicines of Zhejiang Province, Hangzhou Normal University, Hangzhou 311121, China; 3 Drug Discovery, Hangzhou Haolu Pharma Co., Hangzhou 311121, China; linjing32@hotmail.com

**Keywords:** respiratory syncytial virus (RSV), monoclonal antibodies, vaccine, molecule inhibitor

## Abstract

Respiratory syncytial virus (RSV) is a significant viral pathogen that causes respiratory infections in infants, the elderly, and immunocompromised individuals. RSV-related illnesses impose a substantial economic burden worldwide annually. The molecular structure, function, and in vivo interaction mechanisms of RSV have received more comprehensive attention in recent times, and significant progress has been made in developing inhibitors targeting various stages of the RSV replication cycle. These include fusion inhibitors, RSV polymerase inhibitors, and nucleoprotein inhibitors, as well as FDA-approved RSV prophylactic drugs palivizumab and nirsevimab. The research community is hopeful that these developments might provide easier access to knowledge and might spark new ideas for research programs.

## 1. Introduction

Respiratory syncytial virus (RSV) is a significant viral pathogen that causes respiratory infections in infants, the elderly, and immunocompromised individuals, resulting in a substantial disease burden worldwide annually [1]. Two vaccines and a monoclonal antibody therapy have been successfully marketed, and dozens of small-molecule inhibitors are in clinical trials, but effective methods to stop RSV’s virulent spread are still lacking [2]. RSV is an extremely contagious virus [3]. In severe cases, it can lead to fatal complications or repeated infections throughout life. RSV is the leading cause of hospitalization in infants worldwide, with approximately 3.2 million hospitalizations and around 120,000 deaths annually [4].

**Figure 1:**
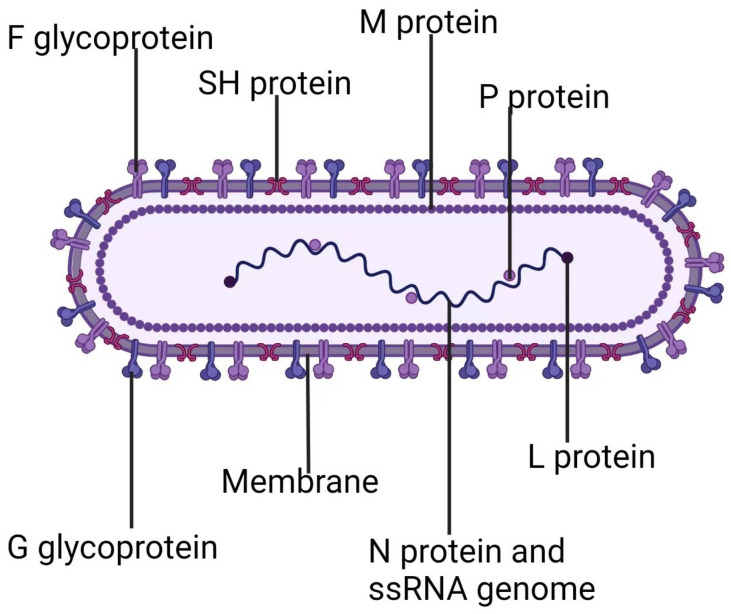
Respiratory syncytial virus. (Created with BioRender.com).

RSV (Figure 1) is an enveloped virus belonging to the family Pneumoviridae. Its genome consists of 10 genes of approximately 15.2 kb in length, which encode 11 proteins, of which 9 are structural proteins and 2 are non-structural proteins [5,6]. The viral membrane anchors the glycoproteins for attachment (G) and fusion (F), as well as the small hydrophobic protein (SH), provided by its lipid envelope. The matrix protein (M) is located on the interior side of the viral membrane. The ribonucleoprotein complex (RNP), which consists of nucleoprotein (N), phosphoprotein (P), and RNA-dependent RNA polymerase (RdRp, L), encapsidates the viral genomic RNA [7,8]. This helical nucleocapsid is resistant to RNase and acts as a scaffold for replication and transcription mediated by the viral polymerase complex [9]. P, N, and L are the essential components for both viral RNA replication and transcription. Transcription involves an additional protein, M2-1, which has a tetrameric structure [10,11]. While N, P, and L alone are adequate for viral RNA genome replication, the involvement of M2-1 is necessary for transcription. M2-1 protein plays a crucial role as a protein that links M and RNP, essential for virion assembly [12]. Two non-structural proteins, NS1 and NS2, which are encoded by the two promoter-proximal genes, promote viral growth by regulating the type I interferon (IFN) activation and response pathway, but their precise targets remain undefined [13,14,15].

RSV infection begins with G-protein binding to cell surface receptors, followed by F-protein-mediated fusion [16]. The nucleocapsid is then released into the cytoplasm. In the cytoplasm, the L, P and M2-1 polymerase complex guide the transcription of the RSV genome, producing primary mRNA transcripts that are translated into viral non-structural and structural proteins [16,17]. The RSV viral genome replicates an antigenome, which serves as the template for genomic RNA synthesis, the N, P and L proteins newly forming an active viral ribonucleoprotein (vRNP) complex [18,19]. The vRNP complex binds to the M2-1 protein, promoting mRNA genome transcription [20]. The glycoprotein complex, comprising the F, G, and SH proteins, confidently associates with vRNP on the cell membrane. The M protein, positioned beneath the membrane, confidently facilitates the subsequent budding of the virus from the apical surface of the lipid raft [21,22,23].

## 2. Potential Treatment Strategies

Thus far, monoclonal antibodies, vaccines, and small molecule drugs have been the primary focus of research on therapeutic measures for RSV. Each strategy has its own advantages and drawbacks, and there is no doubt that more work needs to be done to develop drugs that can successfully pass clinical trials.

### 2.1. Monoclonal Antibodies

Antibodies are an important prophylactic treatment for patients at risk of severe RSV infection. The F protein of RSV is highly conserved among RSV strains and is the primary target of protective neutralizing antibodies [24].

#### 2.1.1. Palizumab

Palizumab, a humanized IgG 1 monoclonal antibody (mAb) developed by MedImmune Inc. in Gaithersburg, MD, USA, a subsidiary of AstraZeneca, was the first commercial humanized monoclonal antibody against RSV. It was approved in 1998 for the prevention of RSV infection [25]. Prophylactic treatment with palizumab has been used for infants born before 29 weeks of gestation and younger than 12 months of age at the start of the RSV season. Prophylactic treatment with palizumab is not recommended for infants born at 29 weeks of gestation or later [26]. Due to its high cost, palizumab is exclusively recommended for children who are at a higher risk of developing severe complications, possess chronic heart and lung disease, or exhibit immunodeficiency [27]. Furthermore, palizumab demonstrated inefficacy in treating persistent RSV infections and did not have significant impact on the overall rate of RSV transmission, as previously believed [28].

#### 2.1.2. Motavizumab

Motavizumab is a second-generation product derived from palivizumab [29]. It has demonstrated higher efficacy in preventing RSV infection, especially in high-risk infants. Motavizumab differs from its predecessor, palivizumab, by 13 amino acid residues. Both mAbs bind to the highly conserved antigenic site A on the RSV fusion (F) glycoprotein, but motavizumab exhibits greater neutralizing activity [30]. Preclinical studies showed that motavizumab reduced viral titers in a cotton rat lung model of RSV infection by 50 to 100 times more than palivizumab [31]. Researchers derived a novel iteration of motavizumab, namely MEDI-557, through substitution of three amino acids within the Fc region of motavizumab (M252Y/S254T/T256E (YTE)) [32]. MEDI-557 displays a 10-fold increase in affinity for FcR, in addition to having a serum half-life four times longer than that of its precursor.

#### 2.1.3. Nirsevimab

With significant progress in mAb screening technology during the past decade, several hundred human antibodies against RSV F proteins have been identified and characterized [24]. Some of these antibodies, specifically tailored to RSV F proteins, exhibit improved efficacy. D25mAb underwent screening that yielded a half-maximal inhibitory concentration (IC_50_) of an RSV value of 2.1 ng/mL. Additionally, D25mAb markedly decreased the ability of RSV to replicate in the cotton rat model. Nirsevimab (MEDI8897) was produced by including the same YTE mutation as in the Fc section of D25mAb. Nirsevimab binds a unique epitope on the pro F protein (not on the post F), so a mAbs are more highly neutralizing. Nirsevimab had greater IgG1 binding to FcRN and more potent anti-degradation characteristics compared to D25mAb [33]. Consequently, it has an extended half-life in serum, with an average half-life of 85 to 117 days, about three times longer than palizumab [34]. Nirsevimab efficiently neutralizes both RSV A and RSV B subtypes. A single dose of nirsevimab decreased the incidence of RSV-induced lower respiratory tract infections and related hospitalizations for 150 days following administration, which is the typical duration of the RSV epidemic season, compared to placebo. There were no significant allergic reactions and nirsevimab demonstrated a favorable safety profile [35]. One significant advanatage of this drug over palizumab is that it requires only one shot, rather than five monthly shots for palizumab, which greatly reduces the cost. It has recently been approved by the U.S. Food and Drug Administration (FDA) [36].

### 2.2. Vaccine

The FDA has licensed two RSV vaccines, and there are several others in development to prevent RSV infection, including live attenuated vaccines, protein/subunit vaccines, vector vaccines, and mRNA vaccines [2]. Developing an RSV vaccine is challenging due to the minimal defense provided by natural RSV infection in healthy adults [37]. All RSV vaccines aim to induce neutralizing antibodies to RSV in the airway mucosa [38].

#### 2.2.1. Lessons from Formalin-Inactivated RSV Vaccines

The development of RSV vaccines was hampered for many years by safety concerns raised by the first trials of an RSV vaccine candidate. In the 1960s, encouraged by the success of the previous formaldehyde inactivated vaccine, researchers conducted several clinical studies soon after the discovery of RSV to evaluate the efficacy of FI-RSV using an alum adjuvant [39,40,41]. The vaccine clearly expressed the post-F conformation of the F protein and was well tolerated. However, upon natural exposure to RSV, many of the previously unexposed infants who had been vaccinated experienced enhanced RSV disease (ERD). About 80% of the vaccine recipients required hospitalization and two fatalities were reported [42]. According to the pathological analysis of deceased children, there was inflammation of the small bronchi and obstruction of the airways with large numbers of neutrophils and eosinophils. Subsequent studies of relevant samples revealed the presence of antibodies with relatively low neutralizing activity [43]. To understand why FI-RSV vaccines not only failed to protect, but actually exacerbated subsequent infection-associated diseases, studies have been conducted using various animal models [44,45,46]. Although there is no definitive conclusion, the FI-RSV vaccine induces poorly neutralized antibodies [43] and little stimulation of CTLs [47], resulting in a higher viral burden and increased immune recruitment. FI-RSV induces an excessive Th2-type immune response that increases mucus secretion, leading to small airway obstruction and increased airway hyper responsiveness, resulting in wheezing [48]. The reasons behind vaccine-induced augmentation are only partially understood, and fear of augmentation has continued to hold back vaccine development for years.

#### 2.2.2. Live Attenuated Vaccines

In infants and children, live attenuated vaccines may have some advantages over non-replicating vaccines. Intranasal immunization with live attenuated vaccines should induce systemic and local immunity, thus preventing infection of the upper and lower respiratory tract. Several live attenuated RSV vaccine candidates have been developed by cold adaptation, chemical mutagenesis and transfer of selected mutations into infectious molecular clones. These candidates have undergone various clinical trials [42].

#### 2.2.3. Protein/Subunit Vaccines

Composed of purified proteins of the pathogen, such as peptides, proteins, or polysaccharides, without the genome of the whole pathogen, subunit vaccines are non-virulent and can be highly effective [49]. Subunit vaccines are a safer option for immunizing immunosuppressed individuals [50]. GlaxoSmithKline released Arexvy (GSK3844766A) in 2020, which is a single-dose subunit vaccine that combines the RSV F protein stabilized in its pre-fusion form and Adjuvant System 01 (AS01E) as an immunostimulant. This vaccine is the first prophylactic vaccine approved by the FDA against RSV-mediated Lower Respiratory Tract Disease (LRTD) and has been available in the U.S. for subjects aged 60 years or older since May 2023. Abrysvo is a highly effective bivalent subunit vaccine candidate that has been developed by Pfizer and was licensed by the FDA in June 2023 for the prevention of RSV-mediated lower respiratory tract disease in subjects aged 60 years or older. The vaccine is administered as a single-dose regimen and is based on the preF protein. Its efficacy and safety have been extensively tested and proven, making it a reliable choice for preventing RSV-mediated lower respiratory tract disease. Abrysvo has been licensed by the European Medicines Agency (EMA) for marketing in Europe as vaccine for the prevention of lower respiratory tract disease in adults aged 60 years or older and in pregnant women in their 24th and 36th weeks of gestational age for infant protection. It is worth noting that Abrysvo is currently the only immunizing agent in the field of RSV prophylaxis that has received market approval for protection in the infant target group [1,2]. DepoVax (DPX)-RSV is a subunit vaccine created at Dalhousie University, which underwent Phase 1 clinical trials [51] This vaccine expresses the RSV N protein and effectively prevents RSV infection in animal models by inducing an RSV N-specific T-cell response. It effectively prevents viral replication during RSV infection without exacerbating clinical signs [52].

### 2.3. Inhibitors

**Figure 2:**
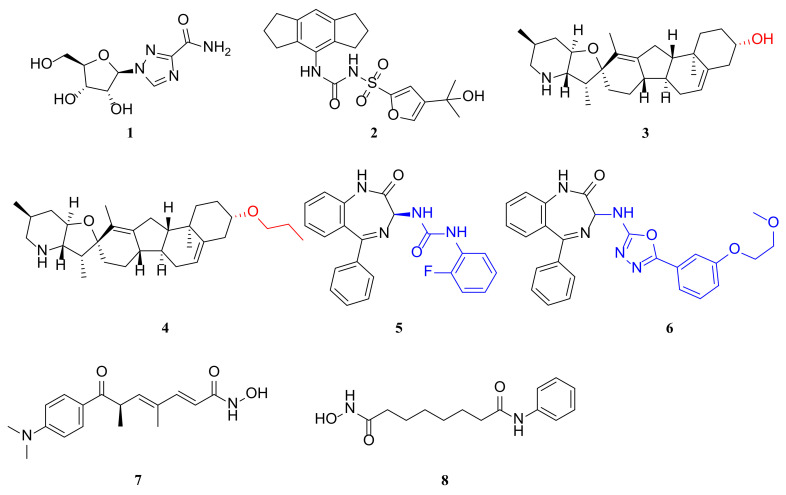
Structure of ribavirin (**1**), MCC950 (**2**), CPM (**3**), A3E (**4**), RSV604 (**5**), EDP-938 (**6**), TSA (**7**) and SAHA (**8**).

Currently, ribavirin (**1**) (Figure 2) is the only small molecule drug approved by the FDA for use against RSV infection. However, controversy over its efficacy, toxicity and the need for special equipment to administer it has limited its use [53]. Thousands of RSV antiviral compounds have been tested for antiviral activity, and some have entered clinical trials. However, no truly effective antivirals have reached the market despite decades of research in this area. New targets and innovative inhibitors of anti-RSV drugs are likely to remain a major area of research. Furthermore, the replication cycle of RSV relies on diverse host proteins and pathways, making the targeting of critical host proteins a promising strategy for the development of effective anti-RSV drugs.

#### 2.3.1. Targeting the NLRP3 Inflammasome

The NLRP3 inflammasome is a protein complex composed of multiple subunits that initiates inflammatory modes of cellular demise and generates the secretion of pro-inflammatory cytokines, namely, IL-1β and IL-18, which significantly contribute to innate immunity [54,55]. The assembly of the NLRP3 inflammasome occurs solely when exposed to pro-inflammatory stimuli, extracellular ATP, pore-forming toxins, or crystals, in conjunction with caspase-1-mediated secretion and pyroptosis of IL-1β and IL-18. The question of how signaling receptors activate gene transcription to facilitate NLRP3 activation remains disputed. Researchers have discovered that RSV infection enhances the expression and activity of both the NLRP3 inflammasome and caspase-1, which are critical for producing IL-1β during RSV infection [56]. Additionally, they identified a mechanism through which the RSV SH protein stimulates the NLRP3 inflammasome. MCC950 (**2**) (Figure 2), a potent and selective NLRP3 inhibitor, effectively hindering IL-1 processing by caspase-1 [57,58].

Present research indicates that the NLRP3 inflammasome plays a crucial part in RSV immunopathology development and subsequent modification of the pulmonary immune environment in the long term. Inhibiting NLRP3 activation during RSV infection in vivo using the small molecule inhibitor MCC950 decreases lung immunopathology, reduces expression of mucus-associated genes, and lessens the production of innate cytokines (IL-1β, IL-33, and CCL2) linked to severe RSV disease. Consequently, it attenuates allergic exacerbations following early RSV infections. These findings offer additional significant proof that the NLRP3 inflammasome is a crucial target for the alleviation of RSV’s immune pathology [58].

#### 2.3.2. Condensate-Hardening Drugs

The replication process of many viruses, including human RSV, takes place in virus-inducing regions known as inclusion bodies (IBs) or viral plasma [59,60]. It has recently been shown that the IBs of negative-stranded RNA viruses can form biomolecular condensates by means of phase separation [61,62]. The steroidal compound cyclopamine and its chemical analogue A3E (**4**) (Figure 2) have been reported to inhibit the replication process of RSV by disrupting and hardening IB condensates. A point mutation in the RSV transcription factor M2-1 blocked the action of cyclopamine and A3E. IB recombination occurs within minutes, suggesting that these molecules directly affect IB fluid properties. A3E and cyclopamine are inhibitors of RSV replication in the lungs of infected mice and are small molecules in the condenser class of drugs with in vivo activity. The data suggest that condensate-hardening drugs can pharmacologically modulate many previously unapproachable targets in viral replication [63].

In infected cells, RSV induces the formation of cytoplasmic IBs, which contain N, P, L, M2-1, and viral genomic RNA, and are the site of viral RNA synthesis. The morphology of IBs suggests that they are condensates [60]. The shape of IBs suggests that they are condensates formed by liquid–liquid phase separation (LLPS). A recent study demonstrated that both N and P can undergo LLPS-driven formation of pseudo-IB condensates in vitro and in cellular settings [64]. These N-P pseudo-IB condensates are non-functional and do not safeguard RNA synthesis, nor do they mirror the intricate nature of IBs in virus-infected cells with multiple compartments. Strikingly, the size and phase organization of RSV inclusion bodies (IBs) closely resemble those of nucleocytoplasmic condensates [65], containing polyphasic IB-associated particles (IBAGs) composed of newly synthesized viral mRNA and M2-1 [66]. The principle of condensates has become significant for subcellular organization. The availability of condensates for drug use is an important consideration in developing antiviral drugs as they can potentially prevent virus replication. In this regard, lytic or hardening drugs can be effective [67].

Researchers examined the effects of two RSV lectin-hardening compounds, A3E and CPM (**3**) (Figure 2), on the replication of RSV in a mouse model [68]. They infected mice with RSV-Luc and treated them with different doses of CPM and A3E twice daily, starting from the day of infection. Treatment with A3E significantly reduced RSV replication in the lungs in a dose-dependent manner. Four days after infection, when the viral load was highest, the lung luminous signal was barely detectable in the treated mice [63]. A3E and CPM also alleviated inflammation and scarring in the lungs of treated animals, which usually caused only mild pathological changes after RSV-A2 infection. Moreover, neither compound could inhibit RSV-Luc in vivo when it expressed a resistant mutation (R151K) in M2-1 (*p* > 0.05), confirming that M2-1 was the main viral protein target of A3E and CPM. These results suggest that the sclerosis of IB agglutinates in infected cells in vitro corresponds to antiviral activity in RSV-infected mice. However, A3E seemed to be slightly less effective than CPM, possibly because of its lower potency in vitro.

#### 2.3.3. Histone Deacetylase (HDAC) Inhibitors

Protein acetylation significantly contributes to host resistance against viral infections through epigenetic modification. Histone deacetylases (HDACs) regulate chromatin structure and gene expression by removing the acetyl group from specific lysine residues on histones. Enzymes not only modify histone targets post-transcriptionally, but also many non-histone targets, such as transcription factors, molecular chaperones, and signaling molecules. These modifications lead to changes in protein stability, protein–protein interactions, and protein–DNA interactions, ultimately controlling a variety of cellular functions with precision and accuracy [69].

Small molecule inhibitors of HDAC, such as TSA (**7**) (i.e., trichostatin A, Figure 2) and SAHA (**8**) (i.e., suberoylanilide hydroxamic acid, Figure 2), have gained attention due to their potential use as anti-cancer drugs. Feng’s group at Nanjing University demonstrated the crucial role of HDACs in RSV replication and their significant impact on virus-associated host defense and inflammatory responses. The study demonstrated that HDAC inhibitors can regulate innate antiviral responses and limit RSV replication. Administering HDAC inhibitors to RSV-infected mice protected them from virus-induced lung injury. The data strongly suggest that HDAC inhibitor treatment limits RSV replication by activating the type I IFN signaling pathway. Administration of HDACs prevents RSV-induced airway inflammation by inhibiting the release of pro-inflammatory cytokines and reducing reactive oxygen species (ROS) production [70].

#### 2.3.4. RSV N Protein Inhibitors

Of the 11 proteins encoded by RSV, N is one of the most highly conserved structural proteins and plays a very important role in the encapsulation of the virus by wrapping around the entire viral RNA genome to form the ribonucleoprotein (RNP) [71]. This multi-functional protein has also been shown to play an important role in viral genome replication, in the transcription of mRNA and in the assembly of the virus [72]. The N protein, with 391 amino acids, binds tightly to the genome and the antigenome to form a helical nucleocapsid, which is the template for RNA synthesis. The N protein expressed in bacteria binds to host RNA and forms a decamer loop, similar to helical nucleocapsid [73]. The N protein also has an antagonistic effect on host innate immune function: N binds to the dsRNA-regulated protein kinase PKR, inhibiting its ability to phosphorylate eIF-2a and inhibiting protein synthesis [74].

RSV604 (**5**) (Figure 2) is a putative inhibitor of the N protein, which is currently undergoing phase 2 clinical trials. However, its molecular mechanism of action remains elusive. The authors examined the cell-line specificity of RSV604 and demonstrated that it can bind directly to N proteins in vitro, indicating that this compound directly targets this class of proteins. The binding affinity of RSV604 to N proteins was not affected by RSV604-resistant mutations in N proteins. In HeLa cells, RSV604 exhibits two distinct mechanisms of action: one that suppresses RSV RNA synthesis and another that reduces viral infectivity. The absence of inhibition of viral RNA synthesis in some cell lines implies that the potency of this inhibitor is cell-type dependent. RSV604 failed to inhibit viral RNA synthesis in RSV sub-genomic replicon cells or in cell-free RNP assays, suggesting that it interfered with a step preceding the formation of the viral replication complex [71].

Optimization of the 1,4-benzodiazepines has led to the discovery of EDP-938 (**6**) (Figure 2) [1], a non-fusion inhibitor of RSV replication that works by modulating the nucleoprotein (N protein) of the virus, preventing it from entering the post-replication phase of the life cycle [75]. In contrast to RSV fusion inhibitors, results from in vitro dose–response studies suggest that EDP-938 is effective even after viral entry into cells. In RSV-infected African green monkeys, bronchoalveolar lavage and nasopharyngeal swab analysis showed that EDP-938 reduced RSV viral load by greater than 90% compared to the control. Pharmacokinetic data from the first-in-human study showed that EDP-938 was rapidly absorbed with little accumulation. The mean half-life ranges from 11 to 18 h across multiple dosing regimens [75]. No significant safety concerns were observed upon repeat oral dosing of up to 600 mg once daily or 300 mg twice daily for 7 days.

#### 2.3.5. RSV F Protein Inhibitors

The RSV F protein is a crucial target for pharmaceutical companies due to its role as the primary neutralization determinant of RSV and its necessity for viral entry and infection. With 574 amino acids, RSV F initiates fusion between the virion and host cell membranes, enabling the release of the viral nucleus capsid contents into the host cell [34].The RSVF protein has two cleavage sites: the first site (KKRKRR; F-137) corresponds to the site found in other paramyxoviruses; the second site (RARR; E-110) is located 27 amino acids upstream [76]. The RSV F0 protein is readily cleaved intracellularly by furin-like proteases and is not a limiting factor for viral infectivity or tropism. The RSVF protein also binds to TLR-4 and initiates signal transduction and innate immune responses [77].

**Figure 3:**
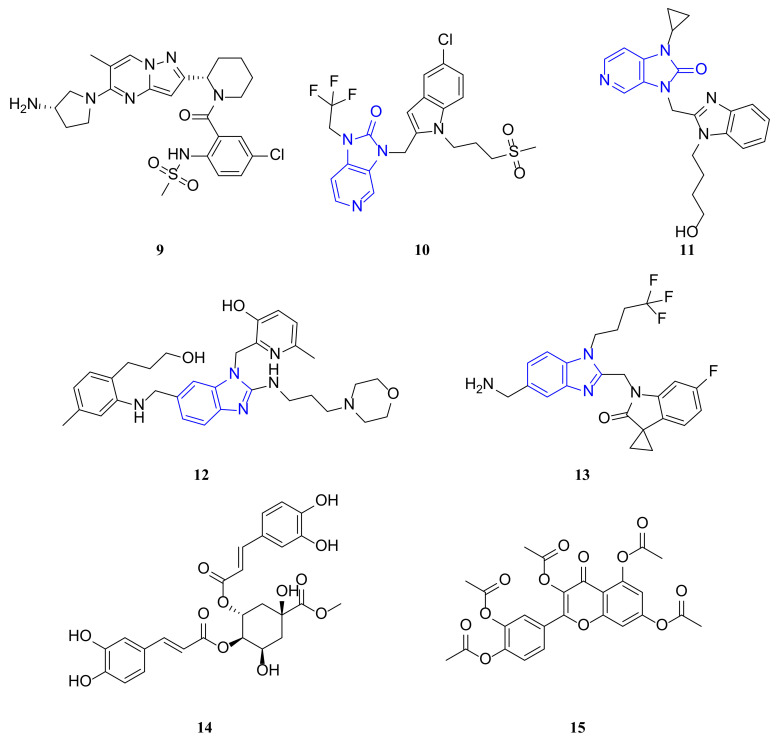
Structures ofGS-5806 (**9**), JNJ-53718678 (**10**), BMS-433771 (**11**), TMC-353121 (**12**), RV521 (**13**), 3,4-DCQAME (**14**) and Quercetin pentaacetate (**15**).

GS-5806 (**9**) (i.e., presatovir, Figure 3) is an inhibitor of RSV F protein [78]. It belongs to a novel class of pyrazolo[1,5-*a*]pyrimidin-2-yl RSV fusion inhibitors, which were identified by Gilead Sciences using high-throughput antiviral screening technology [79]. GS-5806 prevents the fusion of the virus envelope with the host cell membrane, thus preventing entry into the cell [80]. GS-5806 selectively inhibited 75 clinical isolates of RSV subtypes A and B with a mean EC_50_ of 0.43 nM and was discovered by optimizing a novel discovery from a large-scale antiviral screening campaign [78]. Good bioavailability and permeability were observed in the lung tissue of cotton rats. Once-daily oral dosing in healthy adult volunteers infected with RSV resulted in a significant reduction in viral titer and disease severity, with good safety and tolerability [81]. GS-5806 completed a phase 2b clinical trial on adult inpatient RSV cases in April 2017 (NCT02135614) [25].

Johnson and Johnson developed JNJ-53718678 (**10**) (Figure 3), also known as JNJ-678, a fusion inhibitor specific to RSV that binds tightly to the pre-fusion conformation of the RSV F protein, thereby inhibiting RSV infection. In vitro studies have demonstrated that **10** has a strong inhibitory effect on the RSV A2 strain, with an EC_50_ of 480 pM [82]. It is a potent anti-RSV agent in cellular and animal models, with an EC_50_ value of 0.46 nM against recombinant rgRSV224 in HeLa cells, and EC_50_ values ranging from approximately 0.2 to 20 nM against JNJ-53718678 non-recombinant RSV-A and RSV-B strains in a plaque reduction assay. The antiviral efficacy and safety of JNJ-53718678 have been evaluated in clinical studies in healthy adults. Clinical studies in healthy adults evaluated the antiviral efficacy and safety of RSV-A and RSV-B. These findings suggest that the use of RSV-A and RSV-B may be effective in reducing the severity and duration of respiratory tract infections caused by RSV. The studies showed that an average concentration of 0.46 nM reduced viral infection in HeLa cells by 50%. In lamb, mouse, and cotton rat models, it reduced RSV-induced viral entry and lung inflammation, which are two major factors contributing to the severity of RSV disease [25].

BMS-433771 (**11**) (Figure 3) is an orally bioavailable RSV inhibitor that acts by inhibiting membrane fusion induced by the F protein. The compound is active against both groups A and B of RSV with a mean EC_50_ of 20 nM. BMS-433771 is also effective against RSV infection in two rodent models if administered orally prior to infection. The compound has good pharmacokinetic properties while maintaining a good toxicity profile. Therefore, BMS-433771 is well suited for further clinical evaluation in humans. BMS-433771 inhibits lipid membrane fusion during viral entry and late syncytium formation [83]. Biochemical studies have shown that BMS-433771 is a specific inhibitor of membrane fusion induced by the viral F protein. Furthermore, all amino acid changes in drug-resistant viruses are localized to the F1 subunit. This strongly suggests that the F1 polypeptide is a specific molecular target of BMS-433771. BMS-433771 is a novel small molecule antiviral agent suitable for clinical evaluation due to its mechanism of action, in vitro potency, selectivity, and, most importantly, oral efficacy in vivo [84,85].

TMC-353121 (**12**) (Figure 3) is a morpholino-propylamine based benzimidazole RSV fusion inhibitor. A modified derivative of JNJ-2408068 was developed by Johnson and Johnson through molecular modelling and pharmacokinetic studies that identified the key substructures leading to long tissue retention times [86]. TMC-353121 retains the antiviral activity against RSV F of the earlier compound, with a half-life in lung tissue of approximately 14 h and a pEC_50_ = 9.9 nM. In vitro, compound TMC-353121 was found to bind to the HR1 and HR2 regions of the fusion protein F, which inhibited virus–cell fusion and syncytium formation. The prevention of RSV entry into the host cell and produces significant antiviral effects [87]. Treatment of RSV-A2-infected BALB/c mice with compound TMC-353121 showed a notable reduction in viral load in the lungs of mice. The use of compound TMC-353121 in preclinical trials conducted on cotton rats and African green monkeys demonstrated a decrease in viral load and lung inflammation proportional to the administered dosage [87,88].

ReViral in the UK has developed a drug, a fusion inhibitor called RV521 (**13**) (Figure 3), that inhibits the virus fusion phase of the virus by targeting the RSV F protein (EC_50_ = 1 nM) [25]. RV521 demonstrated broad-spectrum activity against multiple RSV subtypes. Specifically, the compound was tested against 14 clinical RSV-A and 10 clinical RSV-B isolates and showed consistent efficacy across subtypes, as evidenced by EC_50_ ranging from 0.1 to 1.4 nM [89]. A recent study on human antiviral efficacy revealed that participants received treatment with an experimental drug either five days after being infected with RSV or upon detection of infection. Both viral load and disease severity were significantly reduced after the drug treatment. Treatment with 350 mg or 200 mg doses of inhibitors resulted in a more rapid reduction of viral load to the detection thresholds. All treatment related adverse events in patients were grade 1 or 2. No subjects discontinued treatment due to adverse events. Only three viral variants were detected and there was no evidence of clinically significant viral resistance. RV521 effectively reduced RSV viral load and disease severity in humans and was well tolerated [90].

In 2018, Tang of Jinan University reported a small molecule, 3,4-DCQAME (**14**) (Figure 3), or 3,4-O-Dicaffeoylquinic acid methyl ester. Compound 3,4-DCQAME inhibits RSV fusion to cell membranes, blocks F protein binding, and reduces RSV-induced pathological changes in mice. This compound can be found in natural products, including those isolated from some traditional Chinese medicines, such as Erycibe obtusifolia [91]. As a derivative of caffeoylquinic acid, 3,4-DCQAME has a unique chemical structure that is distinct from the benzoyl derivative GS-5806, as well as the benzimidazole-based inhibitor BMS-433771. In contrast to these known F-protein inhibitors, compound 3,4-DCQAME may represent a new class of anti-RSV F inhibitors. Treatment of mice with 3,4-DCQAME also inhibits viral infection and growth in lung tissue. These findings provide direct evidence of 3,4-DCQAME’s anti-RSV activity and support its potential as a lead compound for anti-RSV therapy [92].

Quercetin is a notable inhibitor of rhinovirus replication in both in vivo and in vitro settings, due to its antiviral properties. It has a broad range of biological activities, including anti-RSV effects. Although its solubility and stability are less than ideal in lipophilic membrane due to the presence of the -OH groups, its effectiveness in inhibiting rhinovirus replication is well established. Karina Alves Toledo’s group at Universidade Estadual modified quercetin’s structure by acetylating it to obtain quercetin pentaacetate (**15**) (Figure 3), which significantly enhances the anti-RSV action and virucidal activity of quercetin. Quercetin pentaacetate exhibits a stronger interaction with F-proteins, resulting in lower binding energy and better stability, effectively blocking viral adhesion. Its hydrophobic character also facilitates interaction with the protein lumen, further enhancing its efficacy. Quercetin pentaacetate has not yet been proven effective during in vivo infection. However, it is important to note that its EC_50_ falls within a similar range to that of ribavirin [93].

#### 2.3.6. RSV L Protein Inhibitors

The RSV L protein contains 2165 amino acids and is responsible for carrying out all the catalytic activities required for RNA synthesis, mRNA capping and mRNA polyadenylation [72,94,95]. Analysis of RSV mutants has tentatively identified functional regions of L, including the polymerizing structural domain, a putative nucleotide-binding site involved in capping, and residues affecting the efficiency of GE signal recognition. Bioinformatic analysis has shown that the L protein can be divided into six conserved regions (CR I to CR VI) connected by flexible linker regions [96,97]. This suggests that the protein has a modular organization. Although some functions have been specified, the role of each conserved L structural domain in RdRp activity is still incompletely understood [98,99]. Biochemical studies of the RSV and MeV L-proteins confirmed that the L-protein can be split into two distinct fragments that, when co-expressed, are able to restore the biological activity of RdRp by trans-complementation [100]. This finding suggests that the L protein consists of at least two independent structural domains. This multi-domain structure and concentration of several essential enzymatic centers makes L proteins rich in potential drug targets.

**Figure 4:**
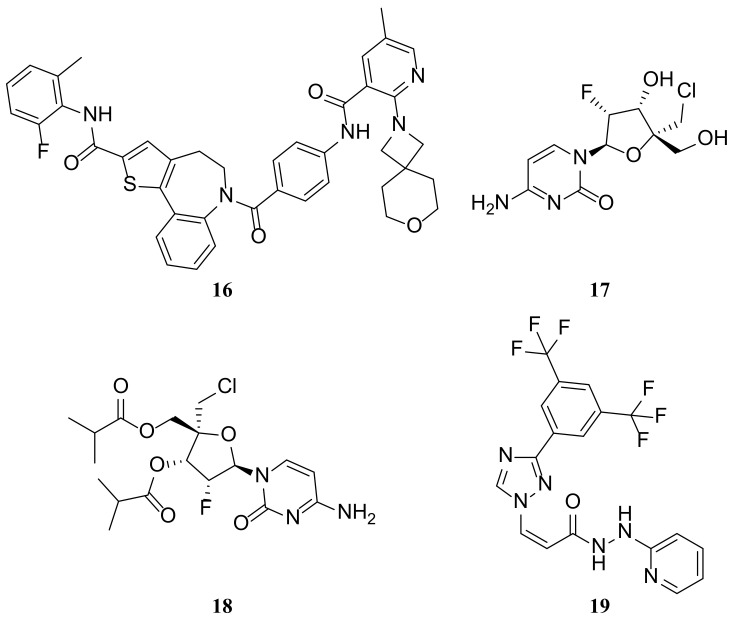
Structures of PC786 (**16**), ALS-8112 (**17**), ALS-8176 (**18**) and KPT-335 (**19**).

PC786 (**16**) (Figure 4) is a highly effective non-nucleoside inhibitor of the RSV L protein polymerase [101]. It is currently being developed as a potential treatment for RSV infection through inhalation. PC786 inhibits viral genomic RNA replication and mRNA transcription by inhibiting RSV polymerase activity. It was effective in preventing RSV polymerase activity in a cell-free enzyme assay, as well as in a mini genome assay conducted in human epithelial type-2 (HEp-2) cells. The IC_50_ levels were 2.1 and 0.5 nM in the two systems [101]. In PC786, the inhibition of viral gene transcription and replication is effectively achieved through RSV A2-derived RNP complexes [101,102]. In vivo, PC786 (2 mg/mL) completely inhibited the viral load in the lungs of BALB/c mice after intratracheal (20 µL) or intranasal (40 µL) administration. In cotton rats, PC786 dose-dependently suppressed RSV titers in lung homogenates. Moreover, at 3.3 and 10 mg/mL (50 µL intranasal), PC786 significantly suppressed RSV titers. PC786 demonstrated a significant and persistent anti-RSV effect on human bronchial epithelial cells. This effect was observed via concentration-dependent inhibition of RSV-A2 viral replication. Treatment with PC786 begun on day 3 after virus inoculation resulted in undetectable viral load by day 6 [103].

The small molecule ALS-8112 (**17**) (Figure 4) is a first-in-class nucleoside analogue prodrug currently undergoing clinical evaluation. The antiviral activity of ALS-8112 is achieved by inhibiting the intracellular RNA polymerase of the RSV using its metabolite ALS-8112-triphosphate. Resistance-associated mutations within the RNA polymerase region of the L gene that encodes the virus are selected by ALS-8112. In biochemical assays, the recombinant RSV polymerase complex recognizes ALS-8112-triphosphate efficiently, leading to the termination of RNA synthesis. ALS-8112-triphosphate did not inhibit the polymerase of the host or unrelated viruses, like hepatitis C virus (HCV). However, some structurally related molecules demonstrate dual inhibition of RSV and HCV. The molecular target of ALS-8112 was identified as the RSVL protein through the selection of resistance-associated mutations in the L gene. ALS-8112-triphosphate stopped RNA synthesis and inhibited RSV polymerase activity by enzyme assay. In support of its specificity for RSV polymerase, ALS-8112-triphosphate had no inhibitory activity against host or HCV polymerase [25].

The poor oral bioavailability of ALS-8112 led to the development of ALS-8176 (**18**) (Figure 4), also known as lumicitabine [104]. This compound undergoes tri-phosphorylation after entering the host cell and competes with cytidine triphosphate to bind to the RSV RdRp. A clinical trial conducted on healthy adults demonstrated that individuals treated with ALS-8176 experienced a 73% to 88% reduction in RSV load in nasal flushes compared to those who received a placebo. The results showed that RSV did not develop resistance to ALS-8176 and that it effectively inhibited RSV replication [105]. In 2018, Janssen completed a Phase I study on infants hospitalized for bronchiolitis, and ALS-8176 is currently undergoing a Phase II clinical trial [106].

#### 2.3.7. RSV M Protein Inhibitors

The 256-amino acid M protein plays key roles in virion morphogenesis [16,72]. The RSV M protein controls viral assembly and egress from respiratory epithelial cells [107]. It is initially located in the nucleus of infected cells during the early stages of the viral life cycle and could cause a slight inhibition of host transcription in the course of RSV infection. Late infection, M translocates to the cytoplasmic inclusion body, becoming a part of the vRNP complex [18]. Nuclear import of M proteins is mediated by importin 1, a nuclear import receptor. Meanwhile, exportin 1 (XPO1) transports M proteins from the nucleus to the cytoplasm. Inhibition of XPO1-mediated nuclear export with LMB leads to decreased virus production and nuclear accumulation of M protein. Furthermore, altering the nuclear export signal (NES) of the M protein results in its nuclear accumulation, and RSV containing M proteins with mutated NES are nonviable [108,109]. It has been suggested that M is the driving force for the assembly of RSV [110,111]. The Oomens group’s recent study found that an RSV M-null mutant exhibited failed RSV viral filament elongation, demonstrating the crucial role of the RSV M protein in driving filamentous particle formation [112].

A novel class of Selective Inhibitors of Nuclear Export (SINE) compounds targeting XPO1-mediated export has shown antitumor and antiviral activity in preclinical and several clinical studies [113,114]. KPT-335 (**19**) (i.e., verdinexor, Figure 4), which has shown broad-spectrum antiviral activity in mouse and ferret model systems against a range of influenza A and B viruses, is being investigated as a potential broad-spectrum treatment for viral diseases [115,116]. As a result, influenza virus replication was reduced and disease incidence decreased. In a previous study conducted as a randomized, double-blind, placebo-controlled, dose-escalation Phase 1 clinical trial in healthy human volunteers, KPT-335 was found to be generally safe and well tolerated, with a similar number and severity of adverse events to placebo. Researchers evaluated the antiviral efficacy of KPT-335 against RSV in an in vitro assay. KPT-335 was shown to inhibit XPO1-mediated nuclear export, decrease RSV replication in vitro, cause nuclear accumulation of RSV M protein and decrease RSV titer, be effective against both RSV A and B strains, and decrease viral replication after prophylactic or therapeutic administration. The inhibition of RSV replication by KPT-335 is due to the combined effects of reduced XPO1 expression, disruption of nuclear export of RSV M proteins and inactivation of the NF-κB signaling pathway. KPT-335 treatment also resulted in the inhibition of pro-inflammatory pathways, which has important implications for the effectiveness of KPT-335 in vivo [16].

### 2.4. Other

RNA interference is a natural biological process. Small interfering RNAs (siRNAs), typically 19–23 nucleotides in length, can degrade mRNA sequences directly and specifically, resulting in a decrease in the expression of the corresponding proteins. ALN-RSV01 is an siRNA that targets the RSV nucleocapsid messenger RNA, inhibiting the formation of the nucleocapsid protein and thus reducing virus replication [117]. In a study of infection in healthy adults, intranasal administration of ALN-RSV01 significantly reduced the prevalence of RSV infection. Additionally, a phase II clinical trial in RSV-infected lung transplant recipients observed the safety and tolerability of this siRNA in reducing the risk of occlusive bronchiolitis obliterans syndrome [118].

## 3. Conclusions

It has been over 60 years since the discovery of RSV, one of the primary causes of respiratory disease globally. Ongoing research is shedding light on the mechanisms RSV employs to invade the host and the roles that its various components play. Research on receptors over the past few decades will undoubtedly provide direction for the development of antibodies and vaccines. Ribavirin, two monoclonal antibodies, and two vaccines have been approved for the prevention and treatment of RSV. Our goal is to develop more affordable and accessible anti-RSV drugs to meet the needs of patients worldwide. Ongoing research in approximately 30 clinical interventions and numerous preclinical candidates shows promise for the development of more effective treatments. Evidence suggests that oxidative stress may contribute to the pathogenesis of RSV-induced acute bronchiolitis obliterans and correlate with disease severity, providing a potential avenue for antiviral drug development. Natural products that have antiviral effects can serve as starting compounds for new drug development. To enhance antiviral efficacy, it is recommended to exploring multiple targets and multi-site inhibitors with synergistic effects to minimize the emergence of drug-resistant mutants of RSV. In addition, to improve the success rate of new-generation anti-RSV drug discovery, it is suggested that new strategies and technologies in medicinal chemistry be widely applied.
